# Sustainability of contrast-enhanced breast imaging: a review of current evidence

**DOI:** 10.1186/s13244-026-02300-w

**Published:** 2026-06-15

**Authors:** Iva Biondić Špoljar, Elisabetta Giannotti

**Affiliations:** 1 Department of Diagnostic and Interventional Radiology, University Hospital Centre Zagreb, Zagreb, Croatia; 2 Cambridge Breast Unit, Cambridge University Hospital NHS Foundation Trust, Cambridge, UK; 3 School of Clinical Medicine, University of Cambridge, Cambridge, UK

**Keywords:** Breast imaging, Sustainability, Contrast-enhanced mammography, Magnetic resonance imaging

## Abstract

**Abstract:**

Breast cancer is the most commonly diagnosed malignancy among women worldwide. Contrast-enhanced imaging is central to diagnosis, staging, and treatment monitoring, yet its increasing use raises important environmental concerns. This review critically compares the sustainability of contrast-enhanced mammography (CEM) and breast magnetic resonance imaging (bMRI), focusing on contrast medium ecotoxicity, energy consumption, data storage, digital infrastructure, patient access, and travel-related emissions. Both iodinated and gadolinium-based contrast media persist in aquatic environments and contribute to water pollution. Although standard wastewater treatment removes a higher proportion of iodinated contrast, the injected dose is substantially larger, resulting in a greater overall environmental load. Gadolinium-based media are used in smaller quantities but are poorly removed by conventional treatment processes and may release toxic free gadolinium ions after excretion. While CEM involves ionizing radiation and a smaller field of view, it consumes markedly less energy per examination, generates smaller data volumes, and can be integrated into existing mammography infrastructure—enhancing accessibility, enabling decentralized deployment, and reducing patient travel. CEM is also faster, more cost-effective, and often preferred by patients due to greater comfort and shorter examination time. In contrast, bMRI, though radiation-free and offering wider anatomical coverage, has a significantly higher energy demand and digital footprint. Overall, CEM demonstrates advantages in environmental, economic, and social sustainability without compromising diagnostic performance in selected clinical indications. Radiology departments can meaningfully reduce healthcare’s carbon footprint by incorporating sustainability principles into modality selection, contrast-media management, and workflow optimization.

**Critical relevance statement:**

CEM and bMRI address similar clinical indications with comparable diagnostic accuracy. CEM offers potential sustainability advantages through lower energy use, smaller data volumes, and easier integration into existing infrastructure, although it involves ionizing radiation, a limited field of view, and a higher environmental load from iodinated contrast. Considering sustainability alongside clinical factors can help radiology departments reduce environmental impact while maintaining high diagnostic standards.

**Key Points:**

- Sustainability should be an integral factor when selecting imaging modalities for breast cancer care.
- Both iodinated and gadolinium-based contrast media show environmental persistence and ecotoxic potential, with iodinated contrast producing a higher total environmental load despite greater removal in wastewater treatment.
- CEM consumes markedly less energy per examination and produces smaller data volumes than bMRI, reducing its digital and carbon footprint.
- CEM can be implemented on existing mammography systems, reducing patient travel, exam time, and costs.

**Graphical Abstract:**

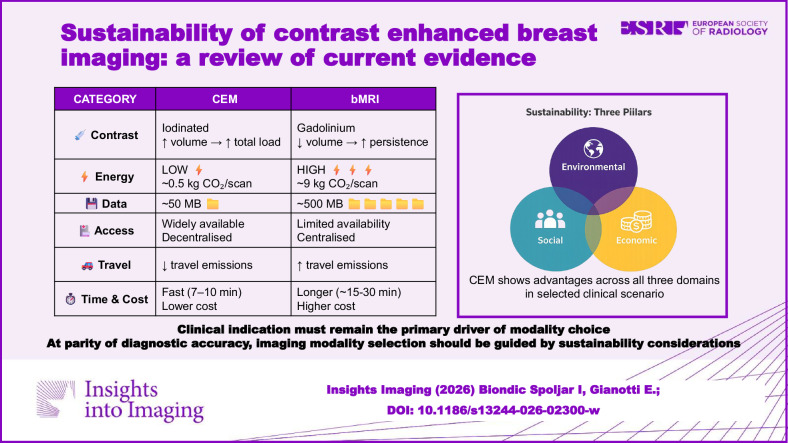

## Introduction

Breast cancer remains the most common malignancy among women, with incidence rates continuing to rise [1]. Contrast-enhanced imaging is essential in modern oncologic care, including breast imaging, playing a critical role in staging, treatment monitoring and cancer surveillance, with also a role as a modality in screening settings. However, it also presents environmental challenges and raises sustainability concerns. Radiology is notably a source of environmental pollution, contributing significantly to the healthcare sector’s global carbon emissions, which account for approximately 4–8.5% of total CO_2_ emissions [2]. Recent literature has increasingly addressed the environmental impact associated with the use of contrast media, energy consumption of imaging modalities, waste production and data storage requirements in radiology—indicating a growing awareness of sustainability issues in medical imaging.

Contrast-enhanced mammography (CEM) is gaining recognition in breast imaging as an alternative contrast using method to breast magnetic resonance imaging (bMRI). CEM received FDA approval in 2011, following results of studies that demonstrated its strong diagnostic performance. CEM has shown comparable effectiveness to bMRI in breast cancer detection and preoperative staging, with improved correlation in tumor size estimation compared to histopathological results [3–6]. Similar accuracy has been demonstrated in evaluating neoadjuvant therapy response and predicting pathologic complete response (pCR) [7, 8], as well as in screening populations with risk higher than the average population [9, 10]. CEM uses ionizing radiation and has a smaller field of view when compared to bMRI.

bMRI has an established role in the detection, evaluation and management of breast cancer. It demonstrates high sensitivity for detecting mammographically occult cancers, particularly in women with dense breast tissue or at increased risk, making it invaluable for both diagnostic workup and supplemental screening strategies. bMRI significantly increases cancer detection and reduces interval cancer rates in women with dense breasts, supporting its integration into risk-adapted screening programs [11–13].

Sustainability encompasses three interconnected pillars: environmental, social, and economic.

In healthcare, environmental sustainability involves reducing carbon emissions and conserving resources; social sustainability ensures equitable access to care and supports the well-being of patients and staff; and economic sustainability focuses on delivering high-quality care efficiently and cost-effectively. Balancing these pillars is essential to providing healthcare that meets present needs without compromising the ability of future generations to do the same.

Given their similar rationale and comparable diagnostic performance in certain diagnostic settings, this paper aims to evaluate and compare sustainability of the two contrast modalities used in breast imaging: CEM and bMRI, through a review of the currently available literature, with a focus on contrast environmental impact, energy consumption, data storage, costs, patient preference and patient travel emissions, while also identifying areas where further research is needed (Table 1).

**Table 1:** Comparison of CEM and bMRI sustainability features

	CEM	bMRI
Contrast medium	Iodinated	Gadolinium
Contrast medium: dose	1.5 mL/kg	0.2 mL/kg
Contrast medium waste in source/tap water	14.2–138.5 ng/L / 3.7–101.3 ng/L	0.3 μg/L–309.4 ng/L
Contrast medium: traditional water waste treatment elimination (%)	38.3%	10%
Energy consumption	8.35 kWh/day	400 kWh/day
Data storage	50 MB	500 MB
Travel emissions	Within breast unit Potential to be done during first visit	Outside breast unit Need for further appointment

## Environmental sustainability: contrast media: persistence and ecotoxicity

For CEM, low-osmolarity iodinated contrast media (ICM) are being used. ICM is the most commonly administered contrast media in radiology, mainly used in CT examination, but also in X-ray images, including fluoroscopy. In many published protocols, iodinated contrast for contrast-enhanced mammography is administered at approximately 1.5 mL/kg, a convention largely extrapolated from CT contrast administration practice and commonly adopted in clinical use. In addition, some centers employ a fixed contrast volume rather than weight-based dosing. However, the iodine concentration of contrast agents (mg I/mL) varies between centers, resulting in differences in total iodine dose for a given volume [14]. Despite their clinical value, there are growing concerns regarding their environmental persistence and ecotoxicity, especially their entry into aquatic ecosystems and potential contribution to climate change, in fact ICM are now frequently detected in surface waters and even in drinking water sources, and growing evidence indicates that their degradation products may be harmful [15].

In individuals with normal renal function, 50% of the administered dose is excreted within 2 h, with prolonged excretion in those with impaired function. Conventional drinking water treatment methods are largely ineffective at removing ICM, allowing these media to enter wastewater systems and ultimately drinking water sources [16]. Hospital effluents are treated together with domestic wastewater, without separate processing [17]. Overall, current treatment processes achieve only limited removal of ICM, with an average efficiency of around 38.3% [18]. This is resulting in ICM being found in water bodies and drinking water in many regions of the world [16, 19] as well as in aquatic fauna, mollusks [20] but also brain and gonads of fish [21]. Evidence shows that 80% of pharmacological effluence within hospital wastewater systems is related to ICM, due to more frequent and widespread use of it in the healthcare system [22]. A Chinese study analyzed ICM concentrations in 25 different drinking water sources, reporting levels of ICM between 14.2–138.5 ng/L in source water and 3.7–101.3 ng/L in tap water [18]. Any water type that is influenced by wastewater effluents, especially in urban areas with diagnostic centers, will most likely show the presence of ICM [16]. ICM can react with disinfectants such as chlorine during water treatment, leading to the formation of iodinated disinfection byproducts—mainly iodoacetic acid and its derivatives—which are more genotoxic and cytotoxic than brominated or chlorinated byproducts [23–25]. Their toxicity arises not from the presence of iodine itself, but from transformation products formed during degradation processes [15]. Environmental factors such as temperature changes and UV exposure can further accelerate ICM breakdown [21]. In wastewater treatment, ICM may undergo biotransformation under aerobic and anaerobic conditions, while advanced oxidation with chlorine, ozone, or chloramines during drinking water treatment can generate particularly harmful iodinated byproducts. The stability and, consequently, persistence of ICM was improved recently, making excreted stable ICM long-lasting pollutants [21]. Due to their high ionic strength and osmolality, ICM are very resistant to biodegradation [15]. Humans are mostly exposed to ICM through drinking water. It is still not fully investigated how water containing ICM affects human health, but there is some evidence of degradation products being toxic.

Contrast media used for bMRI alter proton relaxation times, consequently increasing or reducing signal intensity. Gadolinium is a rare earth element and a non-renewable resource, and gadolinium-based contrast media (GBCM) are the most commonly used for T1-weighted contrast in today’s clinical practice, including breast imaging. They are typically administered intravenously in a dose of 0.2 mL/kg. Free gadolinium ions are toxic, but when chelated, they form stable complexes considered safe for clinical use. To further reduce toxicity, linear chelators have been replaced with macrocyclic ones, which offer greater stability. GBCM are the main source of gadolinium contamination in water bodies. Like ICM, GBCM are excreted unmetabolized via the kidneys, following the same pathway to water bodies. In general, GBCM are renally excreted with plasma elimination half-lives of 1.5 h; however, there is increasing evidence that some of the gadolinium may be retained in the human body, but so far, the exact processes and amounts remain unknown [26].

Most water treatment systems are ineffective at eliminating GBCM, allowing gadolinium chelates to persist in the environment [21]. Standard treatment removes only about 10% of gadolinium [27], as unaltered GBCM are highly mobile and resistant to conventional processes. While advanced oxidation offers some potential for degradation, only reverse osmosis can fully remove these compounds. Macrocyclic GBCM are particularly resistant to breakdown, and UV treatment at the end of the process may produce transformation products with increased health risks [28].

Gadolinium concentrations in surface waters typically range from 0.3 to 80 µg/L, with higher levels in industrialized areas (0.36–26.9 ng/L) and peaks up to 309.4 ng/L near submarine outfalls [29]. Historical data show no gadolinium anomalies before the introduction of GBCM, and current monitoring reveals lower levels on Sundays and Mondays, reflecting reduced MRI activity on weekends [26]. Elevated gadolinium concentrations are consistently detected in lakes and rivers, particularly near hospitals and research centers, but also in rural areas where patients excrete contrast media at home after receiving scans elsewhere [28].

Although GBCM themselves are generally considered non-toxic, free gadolinium ions are harmful. While patients excrete GBCM rapidly, prolonged residence in sewage and the environment increases the likelihood of transformation into toxic free gadolinium through biotic and abiotic processes [26]. Gadolinium contamination has been detected in aquatic flora and fauna worldwide, indicating its entry into the food chain and tap water supplies. For example, a German study found gadolinium in fast food and soft drinks across six cities [30], and another study reported its presence in common ingredients such as flour, rice, and carrots sourced from Europe and Asia [31].

Marine organisms can serve as bioindicators of this pollution. Analysis of great scallop shells from France showed rising gadolinium levels until around 2005, a subsequent decline corresponding to reduced use of linear GBCM, and a slight increase in recent years, likely reflecting the persistence of macrocyclic media [32]. After entering aquatic environments, gadolinium can accumulate in sediments and biota. Although the exact toxicological effects on marine life remain unclear, there is growing concern about long-term bioaccumulation and potential sublethal impacts on reproduction and development. Few studies have addressed these environmental effects due to analytical challenges, leaving significant knowledge gaps regarding GBCM and their impact on aquatic ecosystems.

The environmental impact of contrast media extends beyond water contamination. The entire lifecycle, from production and distribution to use and disposal, contributes to greenhouse gas emissions and therefore to the overall carbon footprint of healthcare [22].

In summary, neither contrast medium is environmentally neutral. Both persist in aquatic environments due to insufficient removal by current standard wastewater treatment methods. ICM are used in larger volumes, resulting in a significantly higher total environmental load. Disinfection byproducts formed during water treatment are of particular concern for human and ecological health. GBCM, although administered in smaller volumes, can release toxic free gadolinium ions, raising concerns about long-term ecological persistence and heavy metal accumulation. There are currently some uncertainties surrounding this topic, and further research is necessary to gain a more comprehensive understanding.

## Environmental sustainability: energy consumption and carbon footprint

Energy consumption and the resulting CO_2_ emission are directly proportional. MRI scanners have shown the highest annual energy consumption, measuring 111,000 kWh/year, followed by CT scanners at 41,000 kWh/year. In comparison, values for X-ray imaging are significantly lower, at about 9500 kWh/year. Ultrasound machines showed the lowest energy consumption, measuring only 760 kWh/year [33]. It has also been shown that a substantial proportion of energy is expended during the idle state [34]. A Canadian study measured and calculated annual greenhouse gas emissions in metric tons per year, showing that X-ray imaging (including standard mammography) produced lower emissions compared to MRI (130 vs. 1327 MTCO_2_/year) [34]. Another study by a group of Australian authors measured CO_2_ emissions per scan, revealing a significant difference between X-ray and MRI scans (0.5 kg/scan vs. 9.2 kg/scan) [35]. Specific data regarding the carbon footprint of mammography, and particularly CEM, is still lacking and requires further investigation. Rossini et al presented data on daily energy consumption for CEM, showing that it requires approximately 8.35 kWh per day, compared to MRI scanners, which can consume up to 400 kWh daily [2, 36].

The energy consumption also varies between scanners, used MRI sequences and gradient systems. Echo-planar imaging and diffusion weighted imaging consume more power in comparison to other pulse sequences. Energy consumption varies by field strength and is higher at 3 T compared to 1.5 T [37].

CEM is a more sustainable technique from the perspective of energy use. Direct comparisons of energy consumption between bMRI and CEM are currently limited, as no published studies have performed modality-specific, head-to-head measurements. Consequently, statements regarding relative energy consumption are inferred from the available literature on MRI systems and mammography units more broadly. While this represents a limitation, the markedly different technical and operational requirements of the two modalities—most notably the need for continuous operation and cooling in MRI—support the conclusion that energy consumption differs substantially, likely by orders of magnitude. In addition, although baseline energy consumption when systems are not actively in use has traditionally been associated with MRI, recent evidence indicates that mammography units may also consume non-negligible energy if not appropriately powered down [36]. Taken together, these observations highlight both the intrinsic differences between modalities and the importance of optimizing operational practices to reduce unnecessary energy use in breast imaging [38].

It needs to be noted that energy is also consumed by the usage of workstations, both for CEM and MRI examination. PACS (Picture archiving and communication system) and RIS (Radiology information system) systems are used for storing patient data and data of imaging related information. The energy consumed by PACS/RIS workstations during active operation is approximately 55 kWh, while during idle periods, it is around 46.8 kWh [39]. It was also shown that workstations left turned on after working hours and during weekends, in one Irish hospital, generated CO_2_ emissions equivalent to the annual emissions of ten passenger cars [40]. An American study demonstrated a 76.3% reduction in energy use by shutting down the workstations when not in use [41].

## Environmental sustainability: data storage and digital infrastructure

Over the last few decades, radiology has largely transitioned from analog to digital systems, with all data now digitized and stored in data storage systems or data centers. Data storage in general contributes nearly the same amount of CO_2_ emissions as the airline industry [42]. It is estimated that a single data center uses as much electricity as approximately 50,000 households [43]. These centers house servers, storage devices, and cooling systems, all of which require a significant amount of energy to operate and maintain. When considering cloud storage, these systems run continuously, while local storage solutions can be switched off when not in use, thereby reducing energy consumption [44]. Imaging modalities vary significantly in their data storage requirements, with MRI requiring the most, while conventional X-ray imaging and ultrasound require much less. The size of the stored data increases proportionally with image quality and the number of obtained images. There is a major difference in file size between CEM and MRI data, with CEM requiring approximately 50 MB per study, while bMRI requires around 500 MB when using standard imaging protocols for both methods. The energy required to store digital images is not negligible. Storing one gigabyte of data consumes between 4 and 7 kWh. As the volume of medical imaging data continues to increase, its contribution to the healthcare energy footprint becomes more significant [45].

## Economic and social sustainability: patient-centered care and travel emission

Sustainability is intended as environmental, social and economic. From a social point of view, patient acceptance and preference for imaging methods should not be overlooked. Results from published surveys have indicated a significantly higher overall preference for CEM over bMRI [46]. CEM can be easily implemented into existing mammography units with a software upgrade and the insertion of a copper filter. Since mammography units are more widely available, upgrading them to perform CEM increases patient access to contrast imaging, while bMRI units remain relatively limited in number [47]. CEM has the potential to be more readily available than bMRI, as it is easier to implement within existing breast imaging workflows. This increased feasibility may lead to broader access and support greater equity in breast cancer care. Increasing accessibility to CEM can help reduce waiting lists for bMRI and also eliminate the need for some patients to travel long distances to other imaging facilities for a bMRI exam. This is especially beneficial in communities where MRI scanners are limited. As CEM units have the potential to be easily distributed in a decentralized manner, contrast-enhanced breast imaging could become more accessible to a greater number of patients, thereby also reducing travel-related emissions. MRI units are typically centralized, resulting in reduced access from rural areas and a higher environmental impact from patient travel. To be mentioned that remote MRI scanning technologies have recently emerged, enabling specialized studies to be conducted at regional hubs and thereby reducing emissions associated with long-distance patient travel from rural and remote areas [37].

Travel and transport are not a negligible factor in the healthcare system’s carbon footprint; in fact, they contribute significantly. CEM has the potential to be offered at the first patient attendance, within the breast unit, reducing the number of patient visits to the hospital and, subsequently, the associated travel emissions. In terms of personnel, CEM requires significantly fewer resources.

From an operational perspective, the time saved with CEM examination is also substantial. A standard MRI appointment per patient varies between institutions and typically lasts up to 30 min, with acquisition time taking 10–20 min, whereas a CEM appointment is shorter and requires only 7–10 min for full examination (preparation and acquisition). Interpretation time for bMRI is longer (3–10 min), while a complete CEM study can usually be interpreted within 1–2 min [7]. Although abbreviated bMRI protocols have been developed and proposed [48], reducing image acquisition and interpretation time, CEM remains overall faster and cheaper. CEM examination is less expensive than either full or abbreviated bMRI protocols, with the total cost being approximately four times lower than that of a full bMRI protocol exam [7]. From an economic perspective, the average price of a new 1.5-T MRI machine—including coils, annual maintenance costs, and MRI safe injector—is at least double the cost of a new mammography unit equipped for CEM, along with its annual maintenance and injector. Furthermore, implementing CEM on already existing mammography units incurs much lower additional costs and space requirements [7].

## Sustainability of artificial intelligence in breast imaging

Artificial intelligence (AI) is increasingly integrated into radiology and particularly breast imaging, bringing transformative capabilities in image analysis, workflow automation, and diagnostic support. While AI holds potential to improve imaging sustainability (such as optimizing imaging protocols, reducing unnecessary procedures, and improving workflow efficiency), its development and deployment are associated with important environmental concerns, and its rapid growth raises critical sustainability challenges. Radiology AI development and deployment are highly energy-intensive due to the computational demands of training and running complex models, often involving deep learning architectures that require powerful hardware and extensive data processing, which together drive substantial energy consumption and greenhouse gas emissions. Research has shown that energy use for training top AI systems can reach millions of kWh, with CO₂ emissions on the order of hundreds of thousands of metric tons from a single training run. These environmental impacts are compounded by the energy demands of data centers and cloud infrastructure needed for storage, which have become significant contributors to the carbon footprint of medical imaging services. A recent scoping review underscores that the current literature on sustainable AI in radiology is still sparse, but identifying key environmental metrics (such as energy consumption and carbon footprint) and emphasizes the need for systematic assessment. Proposed mitigation strategies include the adoption of more energy-efficient model designs, pruning and quantization techniques to reduce computational loads, and incorporating carbon and energy metrics into AI evaluation frameworks. Moreover, there is a call for transparent reporting standards and even the development of eco-labels for AI tools to guide environmentally responsible adoption in clinical settings [49–51].

## Strategies for sustainable breast imaging

**Fig. 1:**
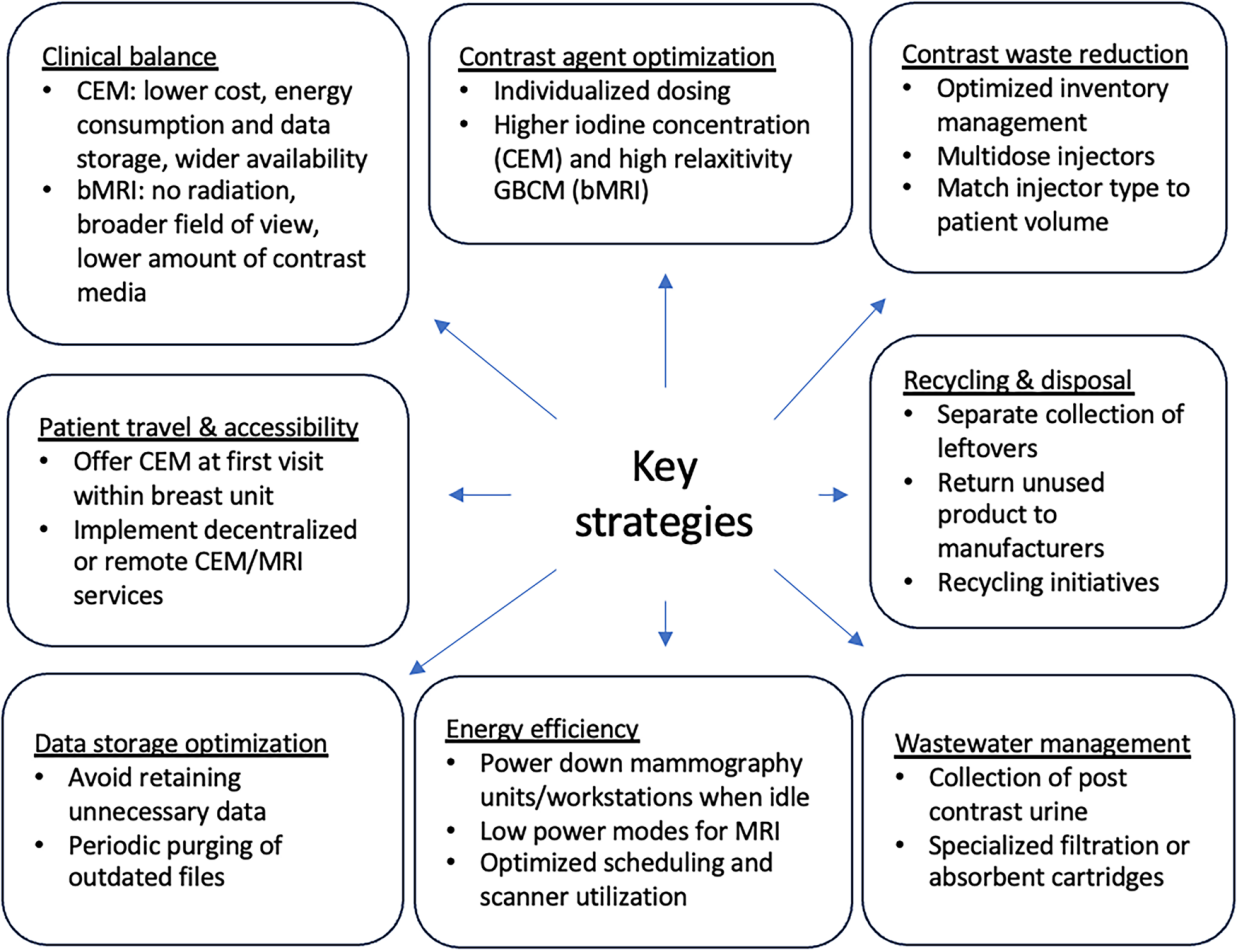
Key strategies for breast contrast imaging sustainability

For a more sustainable future, there is significant potential to implement a range of strategies, including optimizing contrast media use, reducing energy consumption, minimizing waste, and lowering transport-related emissions (Fig. 1).

Regarding the use of contrast media, optimizing the usage and the dose is of crucial importance.

Strategies to reduce contrast use and waste include pre-authorization to avoid unnecessary exams, individualized contrast dosing and the use of advanced post-processing (including AI). The contrast dose could potentially be reduced for both bMRI and CEM: for bMRI through the use of high relaxivity gadolinium media requiring lower doses, and for CEM by using higher iodine concentrations that allow a reduction in contrast volume from 1.5 mL/kg to 1.0 mL/kg, thereby lowering the total iodine dose [52].

Optimizing contrast inventory can reduce contrast waste up to 59% [53], and in high-throughput centers, multidose injectors can virtually eliminate ICM waste. In contrast, in lower-volume settings (< 20 patients per day), their use may actually generate more waste than single-dose systems. This highlights the need to match injector type to patient volume to maximize efficiency and reduce unnecessary contrast waste [54].

Separate collection and incineration of leftovers at hospitals, and recycling of unused contrast material are additional steps toward minimizing environmental pollution [16]. Unused product can be disposed of through hospital waste channels or, if uncontaminated, returned to the manufacturer. The MEGADORE (Medical Gadolinium Recycling) project was established with the goal of creating chemical recycling processes for unused and discarded medical gadolinium [55]. Some of the aforementioned recommendations became unavoidable during the COVID-19 pandemic due to the shortage of ICM caused by global supply chain disruptions [18]. This situation provided a valuable opportunity to reevaluate clinical indications, imaging protocols and the contrast media usage in general, ultimately having a positive environmental impact [56]. As the vast majority of contrast is not treated by standard wastewater treatment, collecting post-contrast urine and specialized wastewater processing techniques have been investigated to prevent contrast medium product from entering the sewage systems. In a recent study by Dekker et al, the majority of patients were willing to use urine bags to help reduce the environmental impact of ICM after a contrast-enhanced CT exam [57]. The Greenwater study in preliminary results achieved median recovery rates of 51% for ICM and 13% for GBCM after collecting the first urine after CT and MRI examinations, presenting a promising avenue for environmental impact reduction in medical imaging [58]. One promising option is to install a dedicated toilet facility within the radiology department that treats excreted contrast media with a special filtration system (iodinated contrast media and gadolinium) through specialized water treatment systems. This approach allows the capture and processing of contrast-contaminated urine before it enters the general wastewater stream, reducing environmental release [59].

In the Netherlands, a cardiology department pilot introduced a separation toilet equipped with absorbent cartridges designed to capture ICM. By completely removing contrast media excreted after procedures [60].

The use of urine bags after ICM administration has been investigated; these bags contain an absorbent pad that converts liquid urine into a solid form, allowing disposal with regular household waste. When incinerated, the iodine contained in the contrast medium is released as naturally occurring iodine and iodine salts, which are already present in the environment [61–63].

The MERK’MAL project in Germany found that using urine bags led to a 20–34% mean and 7–33% median reduction in ICM concentrations in the River Ruhr compared with baseline levels measured at the corresponding wastewater treatment plant, demonstrating that the use of urine bags effectively reduces ICM concentrations in wastewater [64].

Since both imaging equipment and workstations have been identified as major energy consumers, turning off unused mammography machines and workstations during nights and weekends can significantly reduce idle hours and associated energy waste. MRI scanners cannot be fully powered off during normal operations, but they can be placed in a low-power state when not actively imaging [37]. Optimizing patient workflow scheduling can also contribute to minimizing idle time and electricity consumption, ensuring that the scanner operates at a higher utilization rate and that energy is used more efficiently. Furthermore, optimizing data storage by avoiding retention of unnecessary files and regularly purging outdated data helps reduce energy use. In addition, employing a tiered storage architecture can further lower energy consumption by aligning storage resource use with actual data access patterns.

Offering CEM within the breast unit at the patient’s first visit has the potential to streamline the diagnostic pathway, reducing the number of hospital attendances and associated travel-related emissions. Its lower cost and wider availability compared to bMRI also make it feasible and easier to implement in more remote or decentralized settings, increasing equitable access to contrast-enhanced breast imaging while further reducing travel-related environmental impact. It is worth mentioning that remote MRI scanning technologies have also recently emerged, enabling specialized studies to be conducted at regional hubs [37].

While this review focuses on sustainability, it is important to emphasize that clinical considerations must ultimately guide imaging modality selection. From a sustainability perspective, CEM appears advantageous; however, unlike bMRI, CEM involves exposure to ionizing radiation and iodinated contrast, which may restrict its use in certain patient populations. bMRI therefore retains an essential role, particularly for the assessment of breast implants, high-risk gene mutation carriers who are more radiosensitive, and for comprehensive evaluation of the chest wall, inframammary fold, and axillary region, areas where the field of view of CEM is more limited.

## Conclusion

Contrast-enhanced imaging plays a critical role in the diagnosis, staging, and treatment planning of breast cancer, but it also poses significant environmental challenges that must be addressed as part of a broader commitment to sustainable healthcare. Both CEM and bMRI rely on contrast media with documented environmental persistence and potential ecotoxicity. Although standard wastewater treatment removes a larger proportion of iodinated contrast than gadolinium-based media, the injected dose of iodinated contrast is substantially higher. For patients of equivalent weight, the residual environmental load of iodinated contrast is approximately five times greater than that of gadolinium, making it the predominant contributor to contrast-related pollution. Further research is needed to better understand the long-term environmental impact of both contrast media and to develop and implement effective wastewater treatment and mitigation strategies.

Beyond contrast medium, CEM offers several sustainability advantages, including markedly lower energy consumption, reduced data storage requirements, easier integration into existing infrastructure, and the potential to reduce patient travel and associated emissions. Its faster workflow, lower cost, and high patient acceptability further support its role as a sustainable alternative to MRI in appropriate clinical scenarios.

Nonetheless, neither modality is environmentally neutral. Ongoing efforts to optimize dosing strategies, implement wastewater recovery systems, and improve energy efficiency are essential. Future research should focus on elucidating the long-term ecotoxicity of contrast media, developing effective mitigation strategies, and integrating environmental impact assessments into routine clinical decision-making.

## Abbreviations

μg/L: Micrograms per liter
AI: Artificial intelligence
bMRI: Breast magnetic resonance imaging
CEM: Contrast-enhanced mammography
CO_2_: Carbon dioxide
CT: Computed tomography
GBCM: Gadolinium-based contrast media
ICM: Iodinated contrast media
Kg: Kilogram
kWh: Kilowatt-hour
MB: Megabyte
mL/kg: Milliliter per kilogram
ng/L: Nanograms per liter
PACS: Picture archiving and communication system
pCR: Pathologic complete response
RIS: Radiology information system
T: Tesla
TiO_2_: Titanium dioxide
UK: United Kingdom
UV: Ultraviolet
